# The Canadian Assessment of Physical Literacy: methods for children in grades 4 to 6 (8 to 12 years)

**DOI:** 10.1186/s12889-015-2106-6

**Published:** 2015-08-11

**Authors:** Patricia E. Longmuir, Charles Boyer, Meghann Lloyd, Yan Yang, Elena Boiarskaia, Weimo Zhu, Mark S. Tremblay

**Affiliations:** Healthy Active Living and Obesity Research Group, Children’s Hospital of Eastern Ontario Research Institute, Ottawa, Canada; Department of Pediatrics, Faculty of Medicine, University of Ottawa, Ottawa, Canada; Faculty of Health Sciences, University of Ontario Institute of Technology, Oshawa, Canada; Department of Kinesiology and Community Health, University of Illinois at Urbana-Champaign, Champaign, USA

## Abstract

**Background:**

Physical literacy is described as the motivation, confidence, physical competence, knowledge and understanding to value and engage in a physically active lifestyle. As such, it is expected that those who have greater physical literacy would be more likely to obtain the health benefits offered by habitual physical activity. A theoretical model and assessment battery, the Canadian Assessment of Physical Literacy (CAPL), for the assessment of childhood physical literacy had been proposed in theory but validity data were lacking. The purpose of this study was to explore validity evidence for the CAPL among children in grades 4 to 6.

**Methods:**

CAPL validity was evaluated through three analyses that utilized cross-sectional data obtained through local schools in Eastern Ontario, Canada. A confirmatory factor analysis compared the data to the theoretical model. Patterns of association between self-reported age and gender and the CAPL total and domain scores were examined using regression models. Teacher ratings of participants’ knowledge, attitude and physical activity competence were compared to assessment results.

**Results:**

The CAPL was completed by 963 children (55 % female) in grades 4, 5 and 6. Children were 8 to 12 years of age (mean 10.1 years), with 85 % of children approached agreeing to participate. A confirmatory factor analysis using data from 489 children with complete raw scores supported a model with four domains: engagement in physical activity (active and sedentary), physical competence (fitness and motor skill), motivation and confidence, and knowledge and understanding. Raw domain scores followed expected patterns for age and gender, providing evidence for their validity. Interpretive categories, developed from age and gender adjusted normative data, were not associated with age indicating that the CAPL is suitable for use across this age range. Children’s gender was associated with the physical competence, motivation and engagement in physical activity domain scores, indicating that further research is required regarding the gender adjustment of the raw CAPL scores. CAPL domain and total scores were statistically significantly associated with teacher ratings of the child’s motivation, attitudes, fitness, skill and overall physical activity.

**Conclusions:**

CAPL offers a comprehensive assessment of engagement in physical activity, physical competence, motivation and confidence, and knowledge and understanding as components of childhood (grades 4 to 6, 8 to 12 years) physical literacy. Monitoring of these measures enhances our understanding of children’s physical literacy, and assists with the identification of areas where additional supports are required.

## Background

### Importance of physical literacy to child health

Physically active lifestyles are important to children’s health, in the short and long term. Physical literacy, which was defined in a special issue of the Bulletin of the International Council of Sport Science and Physical Education of UNESCO as the motivation, confidence, physical competence, knowledge and understanding to value and engage in a physically active lifestyle [1], can be envisioned as the child’s capability for a healthy active lifestyle [2]. There are four interconnected and essential elements of physical literacy: motivation and confidence (affective domain), physical competence (physical domain), knowledge and understanding (cognitive domain), and engagement in physical activities for life (behavioral domain). This definition, published by the International Physical Literacy Association [3], and these essential elements were recently supported as a joint consensus statement of the leading Canadian non-governmental stakeholders involved in promoting children’s physical activity, including ParticipACTION, Canadian Sport for Life, PHE Canada, Canadian Parks and Recreation Association, Ontario Society of Physical Activity Promoters in Public Health and the Healthy Active Living and Obesity Research Group (cite http://www.participaction.com/canadas-physical-literacy-consensus-statement). The Consensus Statement has been supported by over 1300 physical activity leaders and organizations, including over 50 international leaders from North America (USA, St. Vincent, the Grenadines), South America (Brazil), Europe (United Kingdom, Ireland, Sweden, Italy, Portugal, Slovenia), Asia (Jordan, Qatar, Hong Kong) and Oceania (Australia, New Zealand). A physically literate child will move with confidence and competence across the full spectrum of physical activity settings and opportunities, including activities on land, snow or ice or in the water or air [4]. Conversely, it would be expected that children who are struggling on their physical literacy journey would be less likely to engage in physical activity, increasing their risk for early or accelerated health problems [5]. For optimal health, it is recommended that children perform a minimum of 60 minutes of moderate- to vigorous-intensity physical activity each day, with vigorous and bone-strengthening activities recommended at least 3 days per week [6]. Recent research suggests that very few children achieve this recommended level of daily physical activity [7, 8], and that physical activity begins to decline as early as 3 years of age [9]. The hypoactive lifestyles documented among the majority of children in these studies suggest that at least the behavior component of “physical literacy” may be sub-optimal.

As the concept of physical literacy has gained momentum, so too has the need to be able to monitor it. While there are many established assessments of daily physical activity [10], sedentary behavior [11], motor skill [12], fitness [13], etc., an assessment that encompasses and combines multiple components of physical literacy has not previously been available [2, 14]. A valid, reliable and informative tool that could be used to monitor the four domains within the concept of physical literacy [15] may offer a more comprehensive approach to understanding the factors that influence a physically active lifestyle. A comprehensive physical literacy assessment would also enable population surveillance of the health benefits and risks of engagement in physical activity for life, monitoring of physical literacy educational outcomes, the conduct of research on physical literacy correlates and the evaluation of physical literacy intervention strategies. Enhancing our understanding of children’s engagement in physical activity and aggregated motivation and confidence, knowledge and understanding, and physical competence for physical activity would enable us to better support the development of higher levels of childhood physical literacy.

### Canadian Assessment of Physical Literacy Development

In response to the need for objective data on physical literacy, the Canadian Assessment of Physical Literacy [16] (CAPL) was developed. The goal was to provide a valid, reliable and informative tool for monitoring the physical literacy of Canadian children. In keeping with the internationally accepted definition of physical literacy [3], the CAPL was designed to combine assessments of motivation and confidence, physical competence (health-related fitness and motor skill), knowledge and understanding, and habitual engagement in physical activity (physical activity and sedentary behaviors). Through curricula review and extensive consultations with researchers and practitioners in childhood physical activity and physical education, desired assessment protocols for monitoring physical literacy in children were identified [2, 14]. Existing measures of motivation and confidence, fitness and physical activity were combined with novel assessments of motor skill proficiency [17] and knowledge and understanding. Feasibility of the CAPL components was evaluated through an iterative design and development process. Feasibility was examined in relation to children’s ability to perform the required tasks, as well as administration time and required personnel. In response, the curl-up and push up protocols of musculoskeletal fitness initially recommended were replaced by a static plank hold [18] and the agility and movement skill assessment was revised to fit into a smaller space [17]. Finally, a 3-round Delphi expert review process was completed to establish the validity of the final CAPL model and assessment protocols [19], and to guide development of the CAPL scoring and interpretation procedures. With funding from the RBC Learn to Play Project (http://www.rbc.com/community-sustainability/community/learn-to-play/about-the-rbc-learn-to-play-project.html), Mitacs (www.mitacs.ca), and the Public Health Agency of Canada, the CAPL is currently being used to assess 20,000 children in 12 Canadian provinces and territories. Preliminary data from this national survey have recently been published in the 2015 ParticipACTION Report Card on Physical Activity for Children and Youth [20], with completion of the assessments anticipated by 2017. Although developed for Canadian children, international use of the CAPL is also rapidly expanding [21, 22]. After being introduced at the 25^th^ Pediatric Work Physiology meeting in France [23], international invited presentations about the CAPL have been given in Oceania (29^th^ Australian Council for Health, Physical Education, Recreation International Conference, Adelaide, April 2015), Asia (Southwest University, Chong Qing; Xigang School District, Da Lian; Fujian Normal University, Fuzhou; Shanghai Sports University, Shanghai, China, March 2015) and Europe (University of Jyvaskyla, Jyvaskyla, Finland, November 2012). Based on a recent special issue of the journal of the International Council on Sport Science and Physical Education of UNESCO, the CAPL is unique [16] in its ability to monitor the broad spectrum of characteristics that influence physical literacy.

The primary purpose of this paper was to describe the validity evidence for the CAPL in children in grades 4 to 6, who were 8 to 12 years of age. Secondary aims were to document the CAPL assessment, scoring and score interpretation procedures.

## Methods

### Study design

CAPL validity was investigated using cross-sectional data obtained from children attending local schools in Eastern Ontario, Canada. A confirmatory factor analysis compared the CAPL data to the theoretical model approved through a Delphi process with international experts from America, Europe, Africa, Asia and Oceania [19]. Patterns of association between self-reported age and gender and the CAPL total and domain scores were examined. Teacher ratings of participants’ physical literacy were compared to assessment results. All study procedures were approved by the Research Ethics Board at the Children’s Hospital of Eastern Ontario and the collaborating school boards. Written parental informed consent and child verbal assent were obtained for all participants.

#### Participants

Children were recruited for this study from grade 4, 5, and 6 classes in public schools in Eastern Ontario, Canada. Participating schools were selected to represent a cross-section of communities, including urban, suburban and rural locations. Selected locations were also recruited to represent areas where families typically had low, medium or high levels of income. Recruitment efforts resulted in a convenience sample of 29 schools who agreed to distribute study information to the parents of enrolled children. Research staff answered parent questions in-person or by phone or email. The recruitment target was a minimum of 50 girls and 50 boys in each age or grade group (total n = 300). Two assessment visits occurred at each school, with half of the CAPL protocols completed at each visit. No additional school visits could be made in order to recover missing data for children who were absent or unable to participate on one of the testing days.

### CAPL measurements

Standardized assessments, with established validity and reliability among children 8 to 12 years of age, were identified and incorporated into the CAPL assessment as appropriate. When suitable assessment protocols could not be identified in the published literature, new protocols were developed [17, 19]. For expediency, the data presented in this article refer only to the assessment protocols utilized in validity evaluations of the CAPL. More detailed descriptions of the final CAPL protocols are available in the CAPL manual (available at www.capl-ecsfp.ca).

#### Engagement in Physical Activity

Engagement in physical activity was assessed through measures of physical activity and sedentary behavior. Physical activity was objectively measured, using Digi-Walker pedometers (YAMAX Health & Sports, Inc., San Antonio, Texas), as the number of steps taken each day [24]. Children were instructed to wear the pedometer for 7 full-days and to record the steps taken each day on log sheets. In addition to the number of steps, children indicated what time the pedometer was put on each morning and what time it was taken off for bed at night. Non-wear time was recorded if the child removed the pedometer for anything other than sleeping at night. Children were also asked to self-report the number of days per week that they performed at least 60 minutes of physical activity that was of moderate or vigorous intensity. Sedentary behavior was assessed through self-reported screen time. Children indicated the time spent using computers, televisions and other screen-based devices on school days and on weekends.

#### Physical competence

The physical competence domain within the CAPL was comprised of measures of movement skill and health-related fitness. The Canadian Agility and Movement Skill Assessment [17] was developed as a quick method of monitoring the fundamental (jumping, sliding, catching, throwing, skipping, hopping, and kicking), combined (balance, core stability, coordination, equilibrium, precision) and complex (hand-eye coordination, rhythmic movement, acceleration and deceleration) movement capacities that contribute to physical literacy [25]. The assessment combines scores for the quality of skills performed with completion time, based on the expectation that children with higher levels of physical literacy will be better able to select the optimal combination of speed and skill quality [19]. Measures of health-related fitness included published protocols for muscular endurance (prone plank [18]), muscular strength (handgrip dynamometry [13]), flexibility (sit and reach protocol [13]), cardiorespiratory endurance (PACER shuttle run [26]) and body composition (standing height, body mass, and waist circumference [13]).

#### Knowledge and understanding

The assessment of knowledge and understanding was developed to reflect Canadian provincial curricula for physical and health education in grades 4, 5 and 6. Question content evaluated the children’s understanding of physical activity and sedentary behavior recommendations, awareness of fitness and movement skill parameters and methods for their improvement, perceptions of health, and the use of safety equipment during activity. Children completed the questions using pencil and paper or through an online internet format.

#### Motivation and confidence

Physical activity benefits and barriers were derived from published scales by Garcia and colleagues [27]. The Children’s Self-Perception of Adequacy in and Predilection for Physical Activity (CSAPPA) Scale, developed by Hay and colleagues [28], was used to assess children’s perception of their own ability to be successful in physical activity and their predilection toward physical activity participation.

#### CAPL scoring procedures

**Fig. 1:**
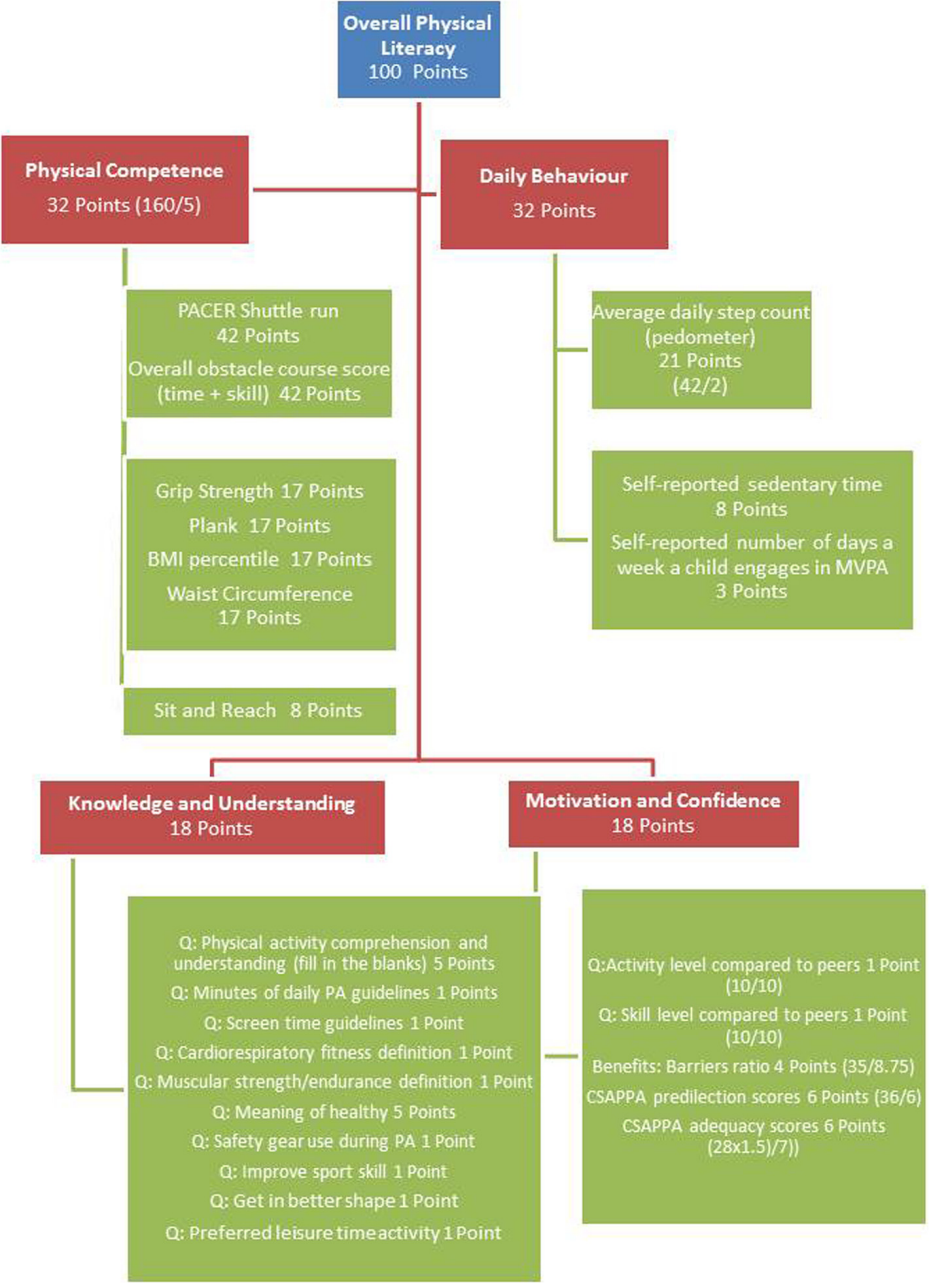
Recommended domains and score weighting for the Canadian Assessment of Physical Literacy

The Delphi Expert Panel recommendations [19] were used to develop the CAPL scoring (Fig. 1) and score interpretation procedures. In keeping with the CAPL model [19] and international definition of physical literacy [3], composite scores can be calculated for the domains of physical competence, motivation and confidence, knowledge and understanding and engagement in physical activity. CAPL total score (100 Points) was a composite calculation where physical activity engagement (32 points) and physical competence (32 points) were weighted equally with less weight assigned to the knowledge and understanding (18 points) and motivation and confidence (18 points) domains. Daily step count was weighted most heavily within physical activity engagement. The agility and movement skill and aerobic fitness assessments were weighted most heavily within the physical competence domain. All questions within the knowledge and understanding and motivation and confidence domains were equally weighted. The Delphi Expert Panel [19] recommended that an algorithm be used to calculate domain scores when one raw score was missing within a domain. It was similarly recommended that an algorithm be used to calculate the total CAPL score if one domain score was missing.

The interpretive categories assigned to the CAPL domain and total score are intended to provide feedback on each child’s progress along the physical literacy journey. Four progressive categories have been identified: beginning, progressing, achieving, exceling. Normative data by age and gender strata were used to define each category. Beginning represents children who are likely to need significant additional support in order to enhance their physical literacy. These children are currently performing below their peers. The progressing category represents the typical performance for Canadian children, which is below the recommended level for a physically active lifestyle. Children who attain the achieving category are performing at a level that is recommended as the minimum associated with optimal health benefits from a physically active lifestyle. The exceling category describes children who are performing substantially above the recommended minimum level. Details regarding the assignment of interpretive categories can be found in the CAPL manual available at www.capl-ecsfp.ca.

### Validation of the CAPL

#### Confirmatory factor analysis

**Fig. 2:**
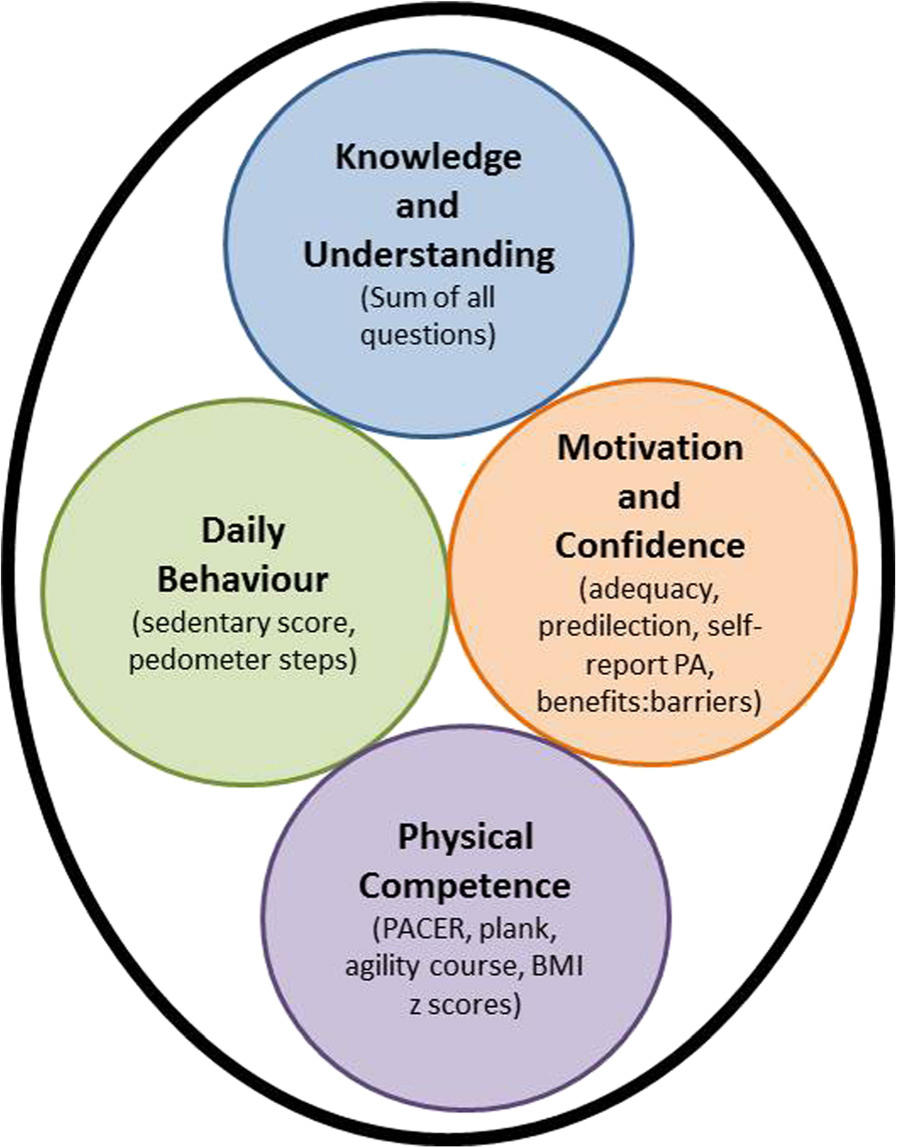
Current theoretical model for the Canadian Assessment of Physical Literacy

The CAPL model (Fig. 2), assessment components and weighting of protocol scores were previously developed through a Delphi process with international experts [19]. A confirmatory factor analysis was performed in order to determine support for the CAPL model based on the assessment results attained by children from local schools in Eastern Ontario, Canada.

#### Age and gender and interpretation of CAPL scores

The association of CAPL total and domain scores with children’s age and gender were examined in relation to expected patterns. Measures of fitness were expected to increase and physical activity to decrease with age, and be higher in boys compared to girls [29]. Interpretive categories for the total CAPL score were summarized for participating children. The association between interpretive categories and self-reported age and gender were examined to ensure that the feedback provided to children would not differ by age or gender, as per the international Delphi Expert Panel recommendations [19].

#### Teacher ratings

Teachers of participating students were asked to provide ratings of each child’s fitness, motor skill, and knowledge and motivation for daily activity. Teacher ratings were provided by either the generalist classroom teacher or the physical education specialist; whoever provided the physical education lessons for the student. Each teacher rating used a scale from 1 (very low) to 10 (very high). Teacher ratings were compared to the CAPL domain and total scores to evaluate the convergent validity of the calculated scores.

### Data quality assurance and control

#### Data management and verification

A valid day of pedometer wear required between 1000 and 30000 steps per day [30, 31] and at least 10 hours of wear time [32, 33]. Three valid days [31] were required to calculate the average daily step count. Data not meeting these standards were marked as missing.

CAPL results for 5 % of the participants were re-entered to assess the accuracy of data entry. Data re-entry was completed approximately 2 weeks after the data collection and initial data entry. In total, 15735 fields were re-entered with 97 total errors giving an error rate of 0.6 %.

#### Personnel training

All assessors involved in this study had post-secondary degrees in physical activity or exercise science. After reviewing the CAPL manual containing detailed instructions for the assessment protocols, all assessors participated in a 1.5 hour practical training session. The lead assessor demonstrated each protocol and then, working with partners, each assessor practiced each protocol. The lead assessor and other assessors observed the protocols being performed and provided suggestions and corrections as required. Practice of the protocols continued until each assessor could accurately administer each protocol and felt comfortable and confident in their own ability to generate reliable results. Additional training sessions were provided for assessors who needed more time to perfect protocol implementation.

### Statistical analyses

#### Confirmatory factor analysis of CAPL protocols

The internationally accepted definition of physical literacy [3] was published during CAPL development. It recognizes four essential elements of physical literacy: motivation and confidence (affective), physical competence (physical), knowledge and understanding (cognitive) and engagement in physical activity (behavior). A confirmatory factor analysis was conducted to determine the fit between this theoretical model and the CAPL assessment results. The threshold for factor loading was set at 0.32 [34].

#### CAPL score distribution

Frequency tabulations were used to describe the distribution of interpretive categories among study participants. The association between interpretive category and self-reported gender was evaluated by chi-square. Regression models examined the associations between: a) age and interpretive category, and b) children’s age and gender and the CAPL total and domain (motivation, knowledge, physical competence, behavior) scores.

#### Teacher ratings

Regression models examined the association between teacher ratings and the CAPL total and domain scores. Ratings of fitness and motor skill were compared to scores for the physical competence domain. Knowledge and motivation teacher ratings were compared to the CAPL knowledge and motivation domain scores, respectively. All teacher ratings were evaluated relative to the total CAPL score.

## Results

### Participants

A total of 963 children (n = 532 female (55 %)) participated from local schools. The mean age of participants was 10.1 ± 1.0 years (range 8 to 12). Self-reported age was missing for 48 children. Estimated age based on grade (grade 4 = 9 years, grade 5 = 10 years, grade 6 = 11 years) replaced these missing values. Recruitment rates were 85 % of eligible children.

### Confirmatory factor analysis

The confirmatory factor analysis was performed on data collected from 489 children (283 female (58 %)) who had complete raw scores for all CAPL protocols. The analysis identified a model with four distinct factors (Table 1). The PACER score, plank score, BMI z-score, and agility and movement skill course score fit within one factor. All of the knowledge questions were summed within a second factor. Adequacy and predilection combined with the benefits:barriers ratio and self-reported activity level score were a third factor. Engagement in physical activity based on pedometer step counts and self-reported sedentary time made up the fourth factor.

**Table 1:** Canadian Assessment of Physical Literacy standardized factor loading matrix for a four factor model (confirmatory factor analysis)

	Physical Competence	Knowledge and Understanding	Motivation and Confidence	Engagement in Physical Activity
PACER	0.73*			
Plank	0.47			
BMI z-Score	0.27			
Agility Course	0.63			
Adequacy			0.85	
Predilection			0.81	
Benefit:Barrier Ratio			0.47	
Activity Compared to Peers			0.62	
Physical Activity Total				0.21
Sedentary				0.19
Sum of Knowledge Items		0.994		

*Factor loading ranges from −1.0 to 1.0. Absolute values closer to 1.0 represent a closer link to the observed item. Threshold of factor loading in this study is 0.32 [34]

For this model, the chi-square (χ^2^) statistic was 98.63 (degrees of freedom (df) = 38, N = 489), with the χ^2^/df ratio having a value of 2.60. The Goodness of Fit Index was 0.96, the Bentler Comparative Fit Index was 0.94, the Bentler-Bonett NFI was 0.91, and the Bentler-Bonett Non-normed Index was 0.91. In addition, the root mean square error of approximation (RMSEA), was .057 (95 % confidence interval: 0.04; 0.07).

#### CAPL score distribution

Domain scores (Table 2) could be successfully calculated for 71 % to 77 % of participants (physical competence: 691/963 or 72 %; knowledge: 687/963 or 71 %; motivation: 740/963 or 77 %; physical activity engagement: 702/963 or 73 %). Half of the children assessed (502/963 or 52 %) obtained complete total CAPL raw scores. Based on the Delphi panel scoring recommendations [19] to use an algorithm to calculate total CAPL score if one domain score was missing, an overall CAPL score could be calculated for an additional 93 children. All children with a missing total CAPL score were missing a value for the physical activity engagement domain plus at least one other domain.

**Table 2:** Canadian Assessment of Physical Literacy total and domain scores by interpretation category

Aggregate Score	Number	Beginning^b^	Progressing^c^	Achieving^d^	Excelling^e^
Total CAPL^a^	403	12 (3 %)	212 (53 %)	117 (29 %)	62 (15 %)
Engagement in physical activity	702	38 (5 %)	335 (48 %)	214 (31 %)	115 (16 %)
Physical Competence	691	44 (6 %)	418 (61 %)	160 (23 %)	69 (10 %)
Motivation & Confidence	740	56 (8 %)	434 (59 %)	232 (31 %)	18 (2 %)
Knowledge & Understanding	687	24 (3 %)	323 (47 %)	231 (34 %)	109 (16 %)

^a^CAPL = Canadian Assessment of Physical Literacy

^b^Beginning = Child is just beginning to develop physical literacy

^c^Progressing = Child is progressing on the physical literacy journey and is performing at a level similar to peers

^d^Achieving = Child is achieving recommended scores indicative of the physical literacy needed to obtain health benefits from a physically active lifestyle

^e^Excelling = Child is exceeding recommended physical literacy levels associated with physical activity health benefits

The interpretive categories for total CAPL score (x^2^ = 7.0, p = 0.07) and knowledge (x^2^ = 5.0, p = 0.17) were not associated with gender. Boys were more likely to be achieving or excelling compared to girls for physical competence (x^2^ = 18.0, p < 0.001), motivation (x^2^ = 18.7, p < 0.001) and engagement in physical activity (x^2^ = 12.4, p = 0.01). Interpretive categories were not associated with age (p > 0.10).

When examining numerical scores rather than the interpretive category, there was a significant age by gender interaction for total CAPL score (p = 0.02, partial eta squared = 0.02 (small effect [35])). Mean scores were similar at 10 years of age and younger (mean difference of 2.2 and 1.1 points (out of maximum 100 points) at ages 9 and 10, respectively). At age 11 years, girls continued to maintain the same mean total score (61 points) but the mean score for boys increased (68 points). At 12 years of age, the mean score for girls had decreased (55 points), while boys maintained the increased mean score (67 points). There was also an age by gender interaction effect for the motivation and confidence domain score (p < 0.01; partial eta squared = 0.01 (small effect [35])). Motivation scores were similar at younger ages and at 12 years of age, but lower among girls at 11 years of age.

Physical competence scores increased with age (1.5 points per year; partial eta squared = 0.11 (moderate effect [35])) and were slightly higher for boys compared to girls (1.2 points; partial eta squared = 0.03 (small effect [35])). Knowledge scores increased with age (0.4 points per year; partial eta squared = 0.02 (small effect [35])). Engagement in physical activity decreased with increasing age (0.7 points/year; partial eta squared = 0.01 (small effect [35])) and was higher among boys (mean difference 1.7 points) compared to girls (partial eta squared = 0.02 (small effect [35])).

#### Teacher ratings

A teacher rating of the child’s physical literacy was available for 555 students. The association between the motivation and confidence domain score (n = 415, 56 % female) and teacher ratings of a child’s predilection towards physical activity was statistically significant, but only about 20 % of the variance was explained (model R^2^ = 0.18, p < 0.001). Teacher ratings of children’s knowledge and attitudes towards physical fitness and motor skill were more related to physical competence (n = 396, 55 % female; model R^2^ = 0.42, p < 0.001). There were little to medium effect sizes for the associations between teacher ratings of the child’s knowledge of physical activity behavior and the knowledge and understanding domain score (n = 378, 58 % female; model R^2^ = 0.03, p = 0.001, η^2^ = 0.04) and the daily behavior domain score (n = 391, 59 % female; model R^2^ = 0.05, p < 0.001, η^2^ = 0.06). When controlling for age and gender, there was a statistically significant association between total CAPL score and overall teacher ratings of a child’s physical activity with about 20 % of the variance explained (n = 196, 59 % female; model *R*^*2*^ = 0.22; p < 0.001).

## Discussion

The CAPL is a comprehensive assessment tool that enables the evaluation of a broad spectrum of components hypothesized to contribute to childhood physical literacy. In our sample of 963 children in grades 4, 5 and 6 (8 to 12 years of age), individual assessment task and domain scores were obtained for over 70 % of our participants. Thus, the CAPL enhances our ability to monitor physical literacy during childhood by enabling simultaneous monitoring of engagement in physical activity, motivation and confidence, physical competence, and knowledge and understanding. Using algorithms to replace a single missing protocol or domain, as recommended by the Delphi panel [19], enabled a total CAPL score and interpretation to be provided to 62 % of study participants.

In this study, data collection for each protocol occurred only once at each participating school. Completion of all CAPL protocols required the children to be present on the two assigned data collection days per school and to complete the pedometer task between the two visits. We expect that completion rates would be much higher in a “real world” setting, where the teacher or coach would have multiple opportunities to ensure that each child completed all of the required protocols. Planning for future research studies utilizing the CAPL should include additional “remedial” assessment dates at each location in order to minimize the missing data due to child illness or absenteeism.

### Confirmatory factor analysis

Results from this study suggest that the CAPL data for children 8 to 12 years of age are largely consistent with the hypothesized CAPL model [19] and the international definition of physical literacy [3]. Consistent with the international definition, our model supported engagement in physical activity (physical activity and sedentary behavior in daily life), physical competence (participants’ fitness and movement skill), motivation and understanding as different types of latent factors. Our confirmatory factor analysis supported a model with four domains, with all measures (χ^2^/df ratio, fit indexes and RMSEA) indicative of good model fit [36, 37]. For the full factor loading (Table 1) there were some standardized coefficient estimates which were somewhat low but most of them were at an acceptable level (above the accepted threshold of 0.32) [34]. These results support the CAPL as an appropriate combination of protocols to represent the four recognized domains of physical literacy (motivation and confidence, knowledge and understanding, physical competence, engagement in physical activity) [3].

#### CAPL score distribution

Completion of the CAPL assessment provides two types of scores. The raw score for each protocol, domain or calculated total score can be used to compare performances within or between individuals or groups. An examination of age and gender associations with the domain scores found the patterns widely recognized in the literature [29]. Knowledge increased significantly with age. Physical competence scores increased with age, and were slightly higher for boys compared to girls. Engagement in physical activity decreased with increasing age, and was higher among boys compared to girls of the same age. Motivation decreased only among girls at 11 years of age, but the small effect size observed suggests that further research is needed to determine the importance and relevance of this association.

It is interesting to note the relationship between changes in motivation and total CAPL score. Motivation decreased for girls at the same age when total CAPL scores first diverged along gender lines (boys increasing, girls remaining the same). Girls motivation then recovered at 12 years of age, even though their total CAPL scores declined. Future research should be completed to confirm this relationship and investigate its importance and relevance to supporting physical literacy among girls. If these results are confirmed, interventions to support higher total CAPL scores among girls 11 years of age may be able to ameliorate the observed decline in motivation. Alternatively, maintaining girls’ motivation for physical literacy at 11 years of age may be helpful in preventing the decrease in total CAPL scores observed among girls who are 12 years of age.

The interpretive categories for each domain and the total CAPL score are used to provide feedback to the participating children and their parents. Categories are based on normative data for children of the same age and gender. The goal is to allow children to complete the same protocols regardless of age or gender, enabling comparisons across time, while at the same time providing realistic feedback to participants regarding their progress along their physical literacy journey. Since interpretive categories are based on normative data for age and gender strata [19], associations between the interpretive categories and age or gender would not be expected. The lack of association between the interpretive categories and age indicates that the normative values assigned to each category are appropriately adjusted across the specified age range (8 to 12 years). Further research is recommended to evaluate the gender differences accommodated by the interpretive categories given that boys are more likely to obtain ratings of achieving or excelling for the physical competence, motivation and engagement in physical activity domains.

#### Teacher ratings

Teacher ratings of a child’s predilection towards physical activity and knowledge and attitudes towards physical fitness and motor skill were moderately associated with the motivation and physical competence domain scores, respectively. Overall CAPL scores were also associated with teacher overall ratings of a child’s physical activity. These results provide some validity evidence in support of the CAPL assessments of motivation and physical competence as well as the combined total CAPL score. In contrast, the associations between teacher ratings of the child’s knowledge about physical activity behavior and both the knowledge and behavior domain scores were statistically significant, but with only a little to moderate effect size (respectively). The limited association between the teacher rating and the behavior domain score is not surprising, as knowledge about physical activity is not necessarily related to increased participation. The very small effect size for the association between the teacher rating of knowledge and the knowledge domain score was particularly surprising. The questionnaire used to assess knowledge was developed based on common elements identified during a review of physical education curricula from all provinces and territories of Canada.

#### Study strengths and limitations

It is important to consider the strengths and weaknesses of this study when interpreting the results obtained. A primary strength of these results is the integrity of the CAPL protocol. It was developed through extensive consultations with experts, teachers and other professionals [16]. The validity and reliability of each protocol within the CAPL battery has been published, with most protocols having been used extensively to assess children. Validity evidence for the CAPL theoretical model and assessment protocols as an whole, was first generated through a Delphi process [19] and then further supported through the confirmatory factor analysis reported in this manuscript.

The large sample of children who participated in the CAPL development and evaluation supports its robustness and the feasibility of CAPL administration in this age group. Over 70 % of participants obtained domain scores, and 62 % achieved a total CAPL score. Alternative evaluation strategies, such as multiple testing dates, should be evaluated to determine whether completion rates can be significantly improved from these levels. Using a two-visit assessment strategy per school, with half of the protocols performed on each day, resulted in 30 % to 40 % of students being unable to complete the full protocol.

Participants were a convenience sample of schools whose principal was willing to participate in this study. Nevertheless, there were similar proportions of males and females and the participating children demonstrated a wide range of skill and fitness levels. Participating schools were found in urban, suburban, and rural locations that represented areas with typically high, medium and low family incomes. Although 85 % of students approached for the study agreed to participate, all children volunteered to participate in the study so the possibility of bias towards children who have higher levels of physical literacy must be considered.

The willingness of children to complete the CAPL testing suggests that the multi-domain nature of the CAPL testing and scoring may buffer participant anxieties associated with performance on any one component. Very few (5 %) children who initially consented to participate subsequently withdrew or refused to complete specific protocols. Injuries were also very rare during testing (<1 %). Generally, teachers were very supportive of the CAPL and recognized the benefits offered by these types of assessments. The length of time to complete the protocols and the inability of a single teacher to conduct all assessments were reported as the potentially problematic aspects of the CAPL protocols for use in schools, although these factors would not significantly restrict use of the CAPL for research.

Additional research is required to develop training materials that will effectively enable the administration of the CAPL by leaders who have not been trained as part of the development team. All of the assessors in this study had undergraduate or graduate degrees in physical activity science, so the ability of individuals without an extensive background in physical literacy to administer the CAPL has yet to be determined. Widespread, international implementation of the CAPL is required in order to acquire sufficient data to establish the validity of the CAPL scoring system, particularly in relation to cultural variations and identifying thresholds for “sufficient” physical literacy.

## Conclusions

The Canadian Assessment of Physical Literacy offers a comprehensive assessment of engagement in physical activity, physical competence, motivation and confidence and knowledge and understanding related to a physically active lifestyle for children in grades 4, 5 and 6 (8 to 12 years of age). Monitoring of these measures enhances our understanding of children’s physical literacy, and assists with the identification of areas where additional supports are required. The ability of individual leaders or those without specialist training in physical activity to administer the CAPL to groups of children has not yet been evaluated.
